# Co‐administration of Omega‐3 Fish Oil and Cannabidiol Restores Cognitive Function and Altered Histoarchitectural changes in the Hippocampus of Sleep‐Deprived Wistar rats through Oxidative Stress Pathway

**DOI:** 10.1002/alz70855_096893

**Published:** 2025-12-23

**Authors:** Gbenga Peter Oderinde, Solomon B Danborno, Uduak Emmanuel Umana, Zainab M Bauchi, Muhammad H Sulaiman

**Affiliations:** ^1^ Ahmadu Bello University Zaria, Zaria, Kaduna, Nigeria; ^2^ Ahmadu Bello University Zaria, Nigeria, Zaria, Kaduna, Nigeria; ^3^ Ahmadu Bello University, Zaria, Kaduna, Nigeria; ^4^ Abubakar Tafawa Balewa University, Bauchi, Bauchi, Nigeria

## Abstract

**Background:**

Sleep deprivation is a prominent problem that is globally prevalent across various age group. Reports have suggested the detrimental effects on various aspects of health, including cognitive function. This study aimed to evaluate the restorative effects of co‐administration of omega‐3 fish oil, and cannabidiol on sleep‐deprived‐induced alterations in the cognitive function, and histoarchitecture of the CA3 region of adult male Wistar rats

**Method:**

Thirty‐five rats were divided into five groups. The control group received 1ml/kg distilled water. Groups II to V were sleep‐deprived for 18 hours daily using the multiple sleep deprivation platform. In addition, Group III was administered 200mg/kg omega‐3 fish oil, Group IV received 10mg/kg cannabidiol while Group V received both 200mg/kg omega‐3 fish oil, and 10mg/kg cannabidiol. Morris water maze was conducted to assess spatial memory. Experimentation and administration lasted for 21 days after which the rats were humanely sacrificed. Blood samples were collected for oxidative stress analysis, the skulls were opened and brains harvested and fixed for histological studies.

**Result:**

The Morris water maze test revealed a significant increase in latency time during the probe test for the sleep‐deprived only group (Group II) compared to the control group. The oxidative stress analysis revealed a significant decrease in Malondialdehyde concentration and an increase in superoxide dismutase, catalase and reduced glutathione in Group V when compared to Group II. Histoarchitectural distortions were observed in the CA3 hippocampal region in Group II however, there were pronounced restorations in Group V.

**Conclusion:**

Co‐administration of omega‐3 fish oil and cannabidiol showed restorative potentials in sleep‐deprived‐induced toxicity in the CA3 region of Wistar rats’ hippocampus by reducing oxidative stress and restoring histoarchitectural distortions.

